# Comparison of intraperitoneal and incisional lidocaine or ropivacaine irrigation for postoperative analgesia in dogs undergoing major abdominal surgeries

**DOI:** 10.1371/journal.pone.0284379

**Published:** 2023-04-13

**Authors:** Federica Alessandra Brioschi, Giuliano Ravasio, Francesco Ferrari, Martina Amari, Federica Di Cesare, Martina Valentini Visentin, Vanessa Rabbogliatti

**Affiliations:** 1 Department of Veterinary Medicine and Animal Sciences, Università Degli Studi Di Milano, Milan, Italy; 2 Centro Veterinario San Martino, Como, Italy; Karolinska Institutet, SWEDEN

## Abstract

This study compared the postoperative analgesic efficacy of intraperitoneal and incisional lidocaine *versus* ropivacaine in dogs undergoing major abdominal surgeries. Dogs randomly received intraperitoneal lidocaine irrigation (4 mg kg^-1^, diluted to 5 ml kg^-1^, L group), ropivacaine (4 mg kg^-1^, diluted to 5 ml kg^-1^, R group) or 0.9% saline (5 ml kg^-1^, C group). Prior to skin closure, dogs received incisional lidocaine 2 mg kg^-1^ (group L), incisional ropivacaine 2 mg kg^-1^ (group R) or incisional saline 0.2 ml kg^-1^ (group C). Pain was assessed at different time points up to 24 hours after extubation, using the Short Form-Glasgow Composite Measure Pain Scale and VAS Scale. In group C, postoperative pain scores were significantly higher than in groups L and R from T0.5 to T6 (p < 0.05). In R group, postoperative pain scores were significantly lower than in groups L and C from T12 to T24 (p < 0.05). Rescue analgesia was administered to 5/11 dogs in L group, 1/10 dogs in R group and 8/10 dogs in C group. Groups L and R experienced a significantly lower postoperative pain during the first 6 hours after extubation, compared with group C. Ropivacaine provided lower postoperative pain scores than lidocaine and saline up to 24 hours after extubation. According to the obtained results, ropivacaine seemed to provide better and longer lasting postoperative analgesia compared with lidocaine. Therefore, intraperitoneal and incisional administration of ropivacaine in dogs undergoing major abdominal surgeries is recommended.

## Introduction

Major abdominal surgical procedures are very common in small animal practice [1]. These procedures are performed under general anaesthesia and are considered to cause moderate-to-severe postoperative abdominal pain [2, 3]. The pain from abdominal surgeries originates from incision, from manipulation of the abdominal viscera and from stretching of the associated ligaments [2]. Appropriate pain management may reduce the recovery time, minimize surgical complications and may also enhance a faster return to normal activities [4].

Intraperitoneal (IP) and incisional (INC) administration of local anaesthetics are simple, safe and low-cost techniques [5] that have been used in human medicine during minimally invasive surgeries [6] and in open abdominal procedures [7, 8]. During the last decade, these non-invasive techniques have gained interest also in small animal practice [9–12]. During IP administration, local anaesthetics are topically applied to the surgical site and the viscera before suturing the abdominal wall [11, 13], while INC local anaesthesia involves an infiltration or topical administration of local anaesthetics on superficial muscles or subcutaneous tissue either before or after surgical incision. Local anaesthetics produce complete blockage of sensory nerve fibers and prevent the development of central sensitization to pain [14]. These agents have also the advantages of being inexpensive and widely available and do not have the adverse effects of systemically administered opioids (sedation, postoperative nausea and impairment of return of gastrointestinal motility) [15].

Lidocaine is a short-acting local anaesthetic, easily adsorbed from the injection site due to its chemical structure [16]. Largely used in humans to provide analgesia following abdominal surgery [17, 18], IP lidocaine has been anecdotally advocated in dogs for treatment of pain related to diseases such as pancreatitis that are very difficult to manage with systemic analgesics [14]. In a canine study, postoperative pain scores indicated the efficacy of IP and INC lidocaine in providing analgesia following ovariohysterectomy [13]. Ropivacaine is an amino-amide local anaesthetic with a similar latency, a longer duration of action and decreased cardiovascular adverse effects when compared to lidocaine [19–21]. Some studies have recently demonstrated that IP ropivacaine has advantages, including prolonged analgesia and low risk of systemic toxicity in dogs [11, 22].

The purpose of this study was to compare the postoperative analgesic efficacy of IP and INC lidocaine *versus* ropivacaine in dogs undergoing major abdominal surgeries. The authors hypothesized that IP and INC lidocaine and ropivacaine would provide effective post-surgical pain relief and that ropivacaine would promote a longer lasting analgesic effect than lidocaine.

## Materials and methods

### Animals

The study protocol was reviewed and approved by the Institutional Ethical Committee for Animal Care at the University of Milan (OPBA_46_2020); owner’s written informed consent was obtained for all the dogs enrolled in the present study. It included thirty-three client-owned dogs, older than 6 months of age and weighing more than 5 kg, of any breed and gender, presented to the Veterinary Teaching Hospital of the University of Milan (Lodi, Italy) for major abdominal surgeries. The health status of dogs at admission was confirmed by physical examination, complete blood cell count, serum biochemical analysis, electrocardiography and echocardiography. Dogs with severe systemic manifestations of disease (American Society of Anaesthesiology class > III) and dogs that were administered analgesics within 72 hours prior to surgery were excluded from the study.

### Study design

This prospective, randomized, blinded clinical study was completed within a 12-month period. All dogs were fasted for 10 hours, and water was withheld for two hours before the beginning of the study. A temperament evaluation was carried out in all dogs using a score ranging from 1 (calm and friendly) to 4 (very excitable and nervous) [23]. Preoperative pain (T0, baseline) was assessed using the Short Form-Glasgow Composite Measure Pain Scale (SF-GCMPS) scoring from 0 (no pain) to 24 (severe pain) [24]. Additionally, a 10 cm visual analogue scale (VAS) with end points labelled as “no pain” (0) and “worst pain imaginable” (10) was used [25]. All dogs were premedicated with dexmedetomidine (5 μg kg^-1^) (Dexdomitor 0.5 mg ml^-1^; Vetoquinol S.r.l., Italy) and methadone (0.3 mg kg^-1^) (Semfortan 10 mg ml^-1^; Dechra Veterinary Products, Italy), mixed in the same syringe and injected into the lumbar epaxial muscles. After 15 minutes, a catheter was aseptically placed in a cephalic vein and anaesthesia was induced with intravenous propofol (Proposure; Merial Italia S.p.A., Italy) titrated to effect to permit the endotracheal intubation. Anaesthesia was maintained with isoflurane (Isoflo; Esteve S.p.A., Italy) in oxygen, dogs were mechanically ventilated using a volume-controlled ventilation mode and respiratory rate was set in order to keep end-tidal carbon dioxide concentration between 35 and 45 mmHg. Lactated Ringer’s solution (Ringer Lattato; Fresenius Kabi, Italy) was administered intravenously at the rate of 3–5 ml kg^-1^ hour^-1^ until extubation. Cefazolin 25 mg kg^-1^ (Cefazolina; Teva S.r.l., Italy) was administered intravenously 20 minutes before surgery. During the intraoperative period, heart rate, invasive blood pressure, haemoglobin oxygen saturation, end-tidal carbon dioxide and body temperature were continuously monitored. In the event of a nociceptive response to surgery, defined as a 20% increase in heart rate and/or mean arterial pressure compared with pre-stimulation values, a fentanyl 1 μg kg^-1^ intravenous bolus (Fentadon; Eurovet Animal Health B.V., The Netherlands) was administered [26]. The number of intraoperative fentanyl boluses given to each dog was recorded. Abdominal surgeries were performed via a midline approach by the same experienced clinical surgeon.

Dogs were randomly (Microsoft Office Excel 2013; Microsoft Corp, Redmond, WA, USA) divided into three groups according to the IP and INC treatments. The groups were L, R and C for lidocaine, ropivacaine and control respectively. Dogs in the L group received IP lidocaine 4 mg kg^-1^ (Lidocaina Cloridrato 1%; Salf S.p.A., Italy). Dogs in the R group received IP ropivacaine 4 mg kg^-1^ (Naropina 1%; AstraZeneca, Italy). Both treatments were of equal volume by diluting drugs with sterile saline to 5 ml kg^-1^; in the L and R groups the final lidocaine and ropivacaine concentration was 0.08%. Dogs in the C group received IP sterile saline 5 ml kg^-1^ (sodium chloride 0.9%; Fresenius Kabi, Italy). Solutions for IP instillation were aseptically prepared by an anaesthesiologist and were administered prior to complete closure of the linea alba, through an intravenous catheter deprived of the inner stylet and inserted at the cranial portion of the midline incision [11]. After complete closure of the linea alba, just prior to skin closure, dogs in the L group received INC irrigation of lidocaine 2 mg kg^-1^ and dogs in the R group received INC ropivacaine 2 mg kg^-1^. Both treatments were of equal volume by diluting drugs with sterile saline to 0.2 ml kg^-1^; in the L and R groups the final lidocaine and ropivacaine concentration was 1%. Dogs in the C group received INC sterile saline 0.2 ml kg^-1^. Time from induction of general anaesthesia to extubation (anaesthesia time) and surgery time were recorded. Times from the end of surgery to extubation (extubation time) and from premedication to extubation (premedication to extubation time) were also registered.

Thirty minutes after extubation, 0.2 mg kg^-1^ meloxicam (Meloxidyl; Ceva, Italy) was subcutaneously administered to dogs. At 0.5, 1, 2, 3, 4, 5, 6, 9, 12, 18 and 24 hours after extubation, sedation and postoperative pain were evaluated by a trained observer who was not aware of treatment allocation. The degree of sedation was assessed with a numerical scoring system ranging from 0 (no sedation) to 3 (profound sedation). Pain assessments were performed using the SF-GCMPS scoring from 0 (no pain) to 24 (severe pain) [24] and VAS scoring from 0 (no pain) to 10 (worst pain imaginable) [25]. Rescue analgesia (methadone 0.2 mg kg^-1^ IM) was administered to dogs with a SF-GCMPS score ≥ 5/20 or ≥ 6/24 and/or a VAS score > 4. Pain scores obtained from dogs receiving the rescue analgesia were excluded from further statistical analysis. Small amounts of food were offered 5 hours after extubation and at any consecutive time points; elapsed time from extubation to first food intake was recorded. A follow-up period of 30 days was planned to evaluate any side effects.

### Statistical analysis

Sample size calculation was performed to identify the number of dogs necessary to detect a difference between treatments in SF-GCMPS, if the C group would have higher scores than the L and R groups (anticipated means, 4.1, 2.3, 2.3 for C, L and R respectively, with 0.5 standard deviation). Based on this calculation, ten dogs per group would provide power of 80% at the α level of 0.05. Mean SF-GCMPS scores and standard deviation were estimated from a pilot study. Statistical analysis was performed using PASW 18.0 (SPSS Inc, Chicago, IL, USA). The normality of data distribution was assessed by a Shapiro-Wilk test at the α = 0.05 level. Data are expressed as mean ± standard deviation and as median and min-max range. Body weight, age, anaesthesia, surgery, extubation and premedication to extubation times were compared among groups using one-way analysis of variance followed by a Tukey’s test. The types of abdominal surgeries performed in each group were compared with Chi-square test. A Kruskal-Wallis test was used to compare ASA status, temperament, the number of fentanyl boluses administered intraoperatively, sedation, SF-GCMPS and VAS scores, time to first food intake and the number of methadone doses administered postoperatively among groups. A Friedman test was used to compare differences in SF-GCMPS and VAS scores over time within each group. Wilcoxon signed-rank tests with a Bonferroni adjustment were also employed as Friedman post hoc. Values for *p* < 0.05 were considered significant.

## Results

Thirty-one out of 33 client-owned dogs met the inclusion criteria and were assigned to the L group (n = 11), R group (n = 10) and C group (n = 10). Two dogs (one in the R group and one in the C group) were excluded from the study after presurgical evaluation, because of biochemical abnormalities. Table 1 summarizes information on the dogs with reference to breed distribution, age, body weight, gender, ASA status, temperament as well as preoperative SF-GCMPS and VAS scores. There were no statistically significant differences between groups regarding age (*p* = 0.96), body weight (*p* = 0.92), ASA status (*p* = 0.65), temperament (*p* = 0.85), preoperative SF-GCMPS (*p* = 0.63) and VAS (*p* = 0.66) scores.

**Table 1:** Breed, age, body weight, gender, ASA status, temperament and preoperative SF-GCMPS and VAS scores of the dogs recruited in L, R and C groups.

Group	Patient	Breed	Age (months)	Body Weight (kg)	Gender	ASA status	Temperament score	Preoperative SF-GCMPS score	Preoperative VAS score
**L**	**1**	Cocker Spaniel	168	13	Female	3	1	6	4
**L**	**2**	Mongrel	132	21	Male	3	2	7	4
**L**	**3**	Bracco Italiano	42	25	Male	2	2	6	4
**L**	**4**	Golden Retriever	36	36	Male	3	1	8	5
**L**	**5**	English Bulldog	96	25	Female	3	1	6	4
**L**	**6**	Bouledogue Francais	98	13	Female	2	2	8	5
**L**	**7**	Labrador Retriever	144	37	Male	2	2	6	3
**L**	**8**	English Bulldog	120	25	Female	2	3	4	2
**L**	**9**	Mongrel	54	37	Female	1	2	2	1
**L**	**10**	Bernese Mountain Dog	64	33	Male	3	1	10	7
**L**	**11**	Cocker Spaniel	60	11	Female	3	1	8	6
**Mean ± standard deviation**			**92 ± 45**	**25 ± 10**		**2 ± 1**	**2 ± 1**	**6 ± 2**	**4 ± 2**
**Median (min-max range)**			**96 (36–168)**	**25 (11–37)**		**3 (1–3)**	**2 (1–3)**	**6 (2–10)**	**4 (1–7)**
**R**	**1**	Bull Terrier	60	21	Male	3	1	8	5
**R**	**2**	American Staffordshire Terrier	48	30	Male	3	2	8	6
**R**	**3**	Labrador Retriever	150	36	Female	3	2	4	2
**R**	**4**	Pinscher	128	6	Male	2	2	4	2
**R**	**5**	Bull Terrier	72	25	Male	2	1	6	4
**R**	**6**	Mongrel	60	22	Female	3	2	7	4
**R**	**7**	German Sheperd	72	44	Male	2	3	8	5
**R**	**8**	Mongrel	110	22	Male	3	2	8	5
**R**	**9**	Mongrel	132	43	Male	3	1	12	7
**R**	**10**	Mongrel	73	21	Male	3	2	12	8
**Mean ± standard deviation**			**91 ± 36**	**27 ± 12**		**3 ± 0.5**	**2 ± 1**	**8 ± 3**	**5 ± 2**
**Median (min-max range)**			**72.5 (48–150)**	**23.5 (6–44)**		**3 (2–3)**	**2 (1–3)**	**8 (4–12)**	**5 (2–8)**
**C**	**1**	Great Dane	18	55	Female	1	2	1	0
**C**	**2**	Pug	48	10	Female	2	2	5	2
**C**	**3**	Mongrel	14	18	Male	3	3	10	6
**C**	**4**	Mongrel	156	22	Female	2	1	6	3
**C**	**5**	English Bulldog	90	29	Female	3	1	6	4
**C**	**6**	Mongrel	132	25	Male	3	1	8	5
**C**	**7**	Fox Terrier	161	7	Female	3	1	9	5
**C**	**8**	English Setter	142	19	Female	3	1	14	8
**C**	**9**	Golden Retriever	125	37	Male	2	2	8	5
**C**	**10**	Labrador Retriever	76	31	Male	2	3	6	4
**Mean ± standard deviation**			**96 ± 55**	**25 ± 14**		**2 ± 1**	**2 ± 1**	**7 ± 3**	**4 ± 2**
**Median (min-max range)**			**107.5 (14–161)**	**23.5 (7–55)**		**2.5 (1–3)**	**1.5 (1–3)**	**7 (1–14)**	**4.5 (0–8)**

Table 2 summarizes information about the type of abdominal surgery each dog underwent and about anaesthesia and surgery times. The statistical analysis detected no differences between groups, with respect to type of abdominal surgery (*p* = 0.45), anaesthesia (*p* = 0.49) and surgery (*p* = 0.32) times. Extubation and premedication to extubation times showed no significant differences between groups (*p* = 0.17 and *p* = 0.33, respectively). The number of fentanyl boluses administered during the intraoperative period did not differ among groups (n = 16, n = 15, n = 23 in L, R and C group respectively, *p* = 0.16).

**Table 2:** Types of major abdominal surgery, anaesthesia, surgery, extubation and premedication to extubation times of the dogs recruited in L, R and C groups.

Group	Patient	Type of Major Abdominal Surgery	Anaesthesia Time (minutes)	Surgery Time (minutes)	Extubation Time (minutes)	Premedication to extubation Time (minutes)
**L**	**1**	Enterotomy	85	58	7	104
**L**	**2**	Splenectomy	131	92	5	156
**L**	**3**	Enterectomy	154	116	13	178
**L**	**4**	Enterotomy	128	103	9	146
**L**	**5**	Cervical Stump Revision	156	114	8	178
**L**	**6**	Enterectomy	86	60	4	106
**L**	**7**	Splenectomy	117	87	8	139
**L**	**8**	Ovariohysterectomy for Pyometra	105	52	5	130
**L**	**9**	Ovarian Remnant Removal	131	100	9	155
**L**	**10**	Gastrotomy + Enterotomy	136	113	9	159
**L**	**11**	Enterectomy	174	109	12	193
**Mean ± standard deviation**			**128 ± 28**	**91 ± 24**	**8 ± 3**	**149 ± 29**
**Median (min-max range)**			**131 (85–174)**	**100 (52–116)**	**8 (4–13)**	**155 (104–193)**
**R**	**1**	Gastrotomy	119	75	9	145
**R**	**2**	Gastrotomy + Enterotomy	125	90	8	156
**R**	**3**	Enterectomy	130	110	4	153
**R**	**4**	Splenectomy	112	75	3	134
**R**	**5**	Enterectomy	120	80	7	147
**R**	**6**	Gastrotomy + Enterotomy	176	140	10	204
**R**	**7**	Prostatic Cyst Omentalization	148	126	9	166
**R**	**8**	Enterectomy	195	150	6	218
**R**	**9**	Intra-abdominal Testicular Neoplasia Removal	117	80	5	145
**R**	**10**	Choledochal Stent and Anastomosis	128	102	4	152
**Mean ± standard deviation**			**137 ± 28**	**103 ± 28**	**7 ± 2**	**162 ± 27**
**Median (min-max range)**			**126.5 (112–195)**	**96 (75–150)**	**6.5 (3–10)**	**152.5 (134–218)**
**C**	**1**	Ovariohysterectomy + Prophylactic Gastropexy	149	123	8	171
**C**	**2**	Ovariohysterectomy for Pyometra	95	52	4	118
**C**	**3**	Gastrotomy	131	94	7	151
**C**	**4**	Splenectomy	115	75	5	137
**C**	**5**	Ovariohysterectomy for Pyometra	152	103	7	170
**C**	**6**	Prophylactic Gastropexy	127	77	7	152
**C**	**7**	Enterotomy	119	87	4	139
**C**	**8**	Splenectomy	83	52	3	101
**C**	**9**	Splenectomy	124	81	9	146
**C**	**10**	Enterectomy	138	115	7	163
**Mean ± standard deviation**			**123 ± 22**	**86 ± 24**	**6 ± 2**	**145 ± 22**
**Median (min-max range)**			**125.5 (83–152)**	**84 (52–123)**	**7 (3–9)**	**148.5 (101–171)**

**Fig 1:**
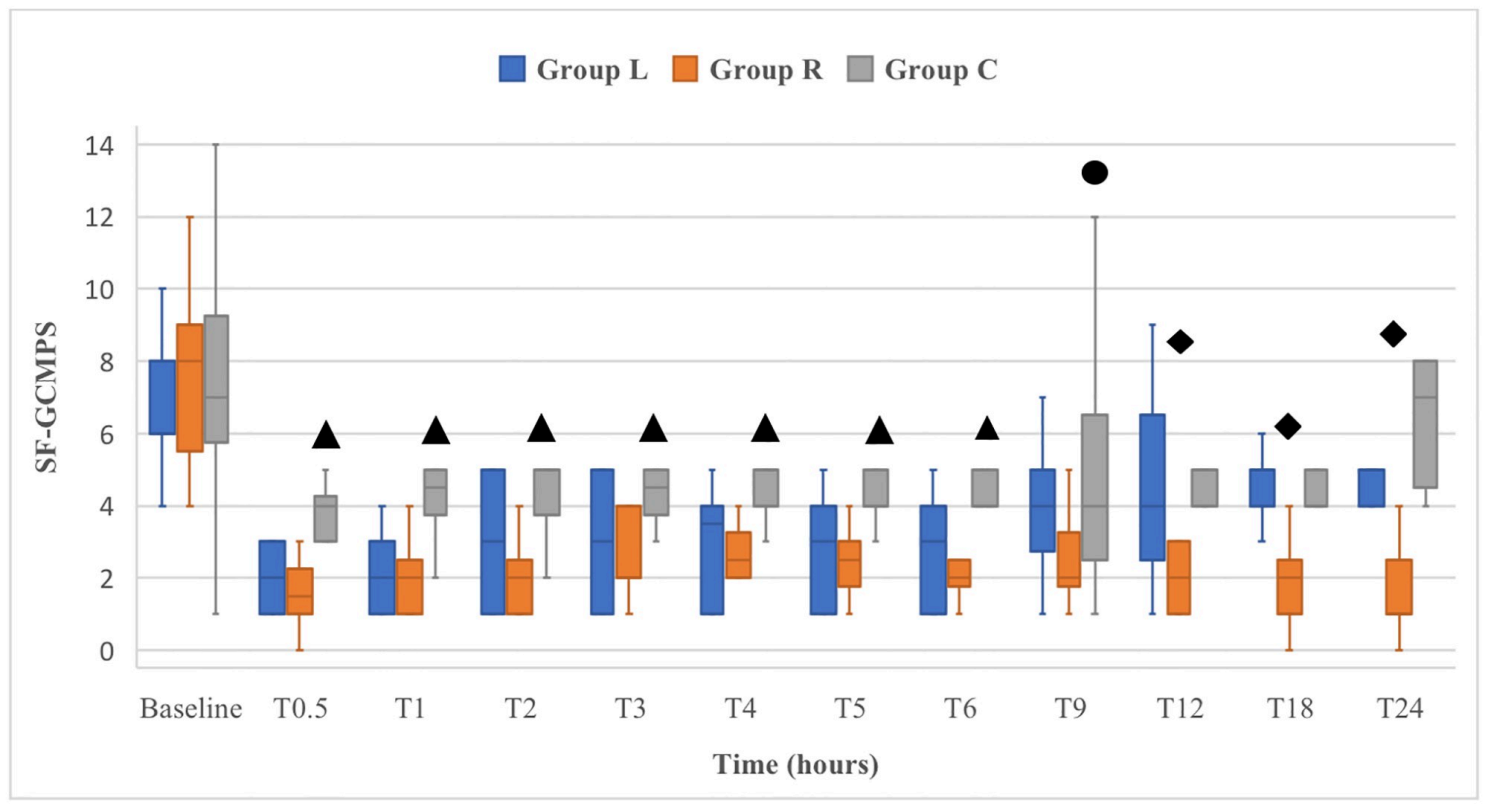
Box-and-whisker plots of the perioperative Short Form-Glasgow Composite Measure Pain Scale (SF-GCMPS) scores in 31 dogs undergoing major abdominal surgeries. Dogs received intraperitoneal and incisional lidocaine (Group L), ropivacaine (Group R) or sterile saline (Group C) at the end of surgery. Dogs were evaluated immediately before surgery (baseline) and from 30 minutes (T0.5) up to 24 hours (T24) after extubation. Each box represents the interquartile range, and the median value is the horizontal line within each box. The upper and lower whiskers represent the upper and lower range of values, respectively. ▲: significantly higher than in groups L and R; ●: significantly higher than in group R; ◆: significantly lower than in groups L and C.

**Fig 2:**
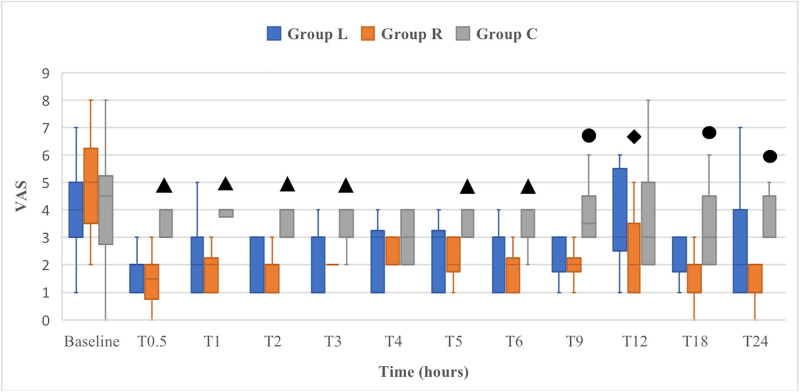
Box-and-whisker plots of the perioperative visual analogue scale (VAS) scores in 31 dogs undergoing major abdominal surgeries. Dogs received intraperitoneal and incisional lidocaine (Group L), ropivacaine (Group R) or sterile saline (Group C) at the end of surgery. Dogs were evaluated immediately before surgery (baseline) and from 30 minutes (T0.5) up to 24 hours (T24) after extubation. Each box represents the interquartile range, and the median value is the horizontal line within each box. The upper and lower whiskers represent the upper and lower range of values, respectively. ▲: significantly higher than in groups L and R; ●: significantly higher than in group R; ◆: significantly lower than in groups L and C.

Postoperative sedation scores did not significantly differ between groups (*p* ≥ 0.05). At T0.5, median sedation score was 2 (1–3), 2 (1–2) and 2 (0–3) in L, R and C group respectively; at T1 median sedation score was 1 (0–2), 1 (0–1) and 1 (0–2) in L, R and C group respectively; at T2, median sedation score was 0 (0–1), 0 (0–1) and 0 (0–1) in L, R and C group respectively; from T3, the sedation score was 0 in all dogs. Postoperative SF-GCMPS scores for dogs in group C were significantly higher compared to those in groups L and R at T0.5, T1, T2, T3, T4, T5 and T6 (*p* < 0.05), but only significantly higher to those in group R at T9 (*p* = 0.033). Dogs in group R had significantly lower postoperative SF-GCMPS scores than those in groups L and C, at T12, T18 and T24 (*p* < 0.05) (Fig 1). Postoperative VAS scores for dogs in group C were significantly higher compared to those in groups L and R at T0.5, T1, T2, T3, T5 and T6, but only significantly higher to those in group R at T9, T18 and T24 (*p* < 0.05). Dogs in group R had significantly lower postoperative VAS scores than those in groups L and C at T12 (*p* < 0.05) (Fig 2).

Postoperative SF-GCMPS scores significantly decreased in groups L and R when compared to baseline scores, at any of the evaluated time points (*p* < 0.05). Postoperative VAS scores significantly decreased in group R when compared to baseline scores, at any of the evaluated time points *(p* < 0.05). In group L, postoperative VAS scores significantly decreased when compared to baseline scores, at any of the evaluated time points (*p* < 0.05), except for T12. The number of dogs that required postoperative methadone was significantly higher in group C than in group R (*p* = 0.002). In L group, one dog received rescue methadone at T1, one dog at T9, two dogs at T12 and one dog at T24. One dog in R group was administered rescue methadone at T12. In C group, one dog received rescue methadone at T3, two dogs at T9, one dog at T12, one dog at T18 and three dogs at T24. Rescue analgesia was administered in 5/11 dogs (45.5%) in L group, 1/10 dogs (10%) in R group and 8/10 dogs (80%) in C group. The time to first food intake was significantly lower in group R than in groups L (*p* = 0.01) and C (*p* = 0.002). Medians (min-max range) of the time of first food intake were 9 (6–12), 5 (5–9) and 9 (6–18) in L, R and C groups, respectively. Vomiting was observed in two dogs at T4 in L group and in one dog at T9 in C group. All dogs recovered without complications and no other adverse effects were observed during the 30-day follow-up period.

## Discussion

Intraperitoneal anesthesia is an inexpensive, simple, and safe method for controlling intraoperative and postoperative pain during abdominal surgery in human patients [5]. Results of the present study showed that dogs that received IP and INC lidocaine and ropivacaine (IP: 4 mg kg^-1^, diluted to 5 ml kg^-1^; INC: 2 mg kg^-1^, diluted to 0.2 ml kg^-1^) at the end of major abdominal surgeries recorded significantly lower postoperative pain scores when compared to baseline values. Dogs in these two groups experienced significantly less postoperative pain (lower SF-GCMPS and VAS scores) during the first 6 hours after extubation, compared to those in the control group. Administration of IP and INC ropivacaine provided lower postoperative pain scores than lidocaine and saline up to 24 hours after extubation, reduced the need for rescue analgesia and promoting early onset of food intake.

In veterinary medicine, pain after major abdominal surgeries is a multifactorial process that includes visceral and somatic pain, from the incision of the abdominal wall, the distension of the peritoneum, stretching of the associate ligaments and traction of nerves and blood vessels [27]. Postoperative pain can cause a decrease in food intake, depression of respiratory function and central sensitization to noxious stimuli that can lead to the development of maladaptive pain [28]. Therefore, to manage postoperative pain after this type of surgery, a multimodal approach is required; in small animals practice this involves the use of opioids, non-steroidal anti-inflammatory drugs and local anaesthetics [29]. The main advantage of using local anaesthetics is that they produce complete blockage of sensory nerve fibers and that they do not cause the adverse effects of opioids and non-steroidal anti-inflammatory drugs, that may reduce the quality of recovery and delay discharge from hospital [27].

Among different regional analgesic techniques, IP and INC administration of local anaesthetics are simple and inexpensive methods that reduce early postoperative analgesic requirements, time to first-intervention analgesia and pain scores after abdominal surgery in humans [7, 30]. The topical application of local anesthetics to the incisional site, the viscera and to the peritoneum exhibits an analgesic effect by blocking nociception from the area of tissue damage; the systemic absorption of local anesthetics through the peritoneal surface may also play a role in the analgesic effect by attenuating nociception both in human [7, 30, 31] and veterinary medicine [32]. Furthermore, local anesthetics have anti-inflammatory actions. A proinflammatory cytokine cascade in the peritoneal cavity, with direct action on the visceral afferents and the vagus as major vehicle, is a feasible contributor to postoperative visceral pain. By using IP and INC local anesthetics, it may be possible to modulate somatic, visceral and peritoneal signaling to the brain, thereby attenuating the metabolic impact of surgery [33].

To the best of our knowledge, no studies evaluating the combined use of IP and INC local anaesthesia during major abdominal surgeries and quantification of its postoperative analgesic effect have been described in companion animals. In dogs, numerous studies have evaluated the effectiveness of IP and INC administration of local anaesthetics for pain relief after ovariohysterectomy and have provided variable results probably due to differences in site and timing (preoperatively or postoperatively) of administration and differences in local anaesthetic doses, concentrations and volumes of injection. Findings of a previous study suggest a possible efficacy of IP and INC lidocaine for treatment of postoperative pain in dogs undergoing ovariohysterectomy; in fact, dogs had lower pain scores at 0.5 hours post-extubation and receive less rescue analgesics than dogs that received IP and INC saline [13]. However, results of another study showed no benefit of IP lidocaine for postoperative pain management after ovariohysterectomy, compared to a group of placebo-treated dogs [34]. Intraperitoneal 0.5% ropivacaine (3 mg kg^-1^), administered in combination with morphine and carprofen, provided postoperative analgesia for 6 hours after extubation in dogs undergoing ovariohysterectomy [11]. However, postoperative SF-GCMPS and VAS scores were statistically similar in dogs that received a placebo [22]. One of the factors that might contribute to failure of IP local anaesthesia for postoperative pain management after abdominal surgeries may be related to inadequate distribution of local anaesthetics throughout the visceral and peritoneal surface. In fact, achieving an even distribution of local anesthetics into the tissues of a surgical site can sometimes be technically difficult and often result in “patchy” analgesia [29]. In contrast, higher volumes should provide a uniform spread of local anaesthetics throughout the peritoneal cavity and thus may be beneficial to improve pain relief after major abdominal surgeries. The volume of IP solution (5 ml kg^-1^) administered in the present study was larger than those previously reported in veterinary literature [11, 13, 22, 34] and it was determined by the experience of anaesthetists and surgeons at the Veterinary Teaching Hospital of the University of Milan, with the goal of providing adequate exposure to the whole visceral and peritoneal surfaces while limiting the risk of leakage from the abdominal cavity during surgery. This large volume was chosen after a pilot study that aimed to compare different volumes; the volume of 5 ml kg^-1^ was the one that ensured the best distribution of the anaesthetic solution in the abdominal cavity with no loss of it during instillation. Furthermore, it is clearly demonstrated that visceral and parietal peritoneum exposure to room air during abdominal surgery promotes local early inflammatory responses [35], probably concurring to the amplification of the nociceptive stimulus. In this study, the large volume (5 ml kg^-1^) in which the local anesthetics were diluted was probably evenly distributed over the entire peritoneal surface, contributing to limit the painful stimuli resulting from general peritoneal cavity inflammation, and not only of that deriving from the area manipulated by the surgeon. Among the multiple factors affecting the postoperative analgesic efficacy of IP and INC instillation, the concentration of the administered local anaesthetic may be important. A previous human study reported a more efficacious sensory block with high-volume low-concentration compared to low-volume high-concentration levobupivacaine in brachial plexus block [36]. High-volume low-concentration IP bupivacaine significantly increased postoperative duration of analgesia and reduced opioids requirement in humans undergoing laparoscopic cholecystectomy [37]. The low-concentration IP lidocaine and ropivacaine (0.08%) used in the present study had not previously reported in dogs. However, tumescent local anaesthesia with 0.05% ropivacaine provided adequate analgesia in dogs undergoing mastectomy [38].

Solutions for IP instillation were administered prior to complete closure of the linea alba so that the surgeon could confirm the distribution at the surgical site and that there was no leakage from the abdominal cavity. Local instillation of large volumes of local anesthetic may increase the risk of their systemic absorption and side effects in people [39], but no clinically relevant adverse consequences or signs of toxicity were noted during the postoperative period in dogs included in the present study. Two dogs in L group and one dog in C group experienced vomiting. A previous study reported a reduced gastrointestinal transit time and an increased occurrence in nausea and vomiting in dogs receiving a lidocaine constant rate infusion at 0.05 mg kg^-1^ min^-1^ [40]. However, in the present study the incidence of this complication was minimal, and no additional supportive care was required. Also, doses administered for lidocaine and ropivacaine were 4 mg kg^-1^ and 2 mg kg^-1^, by IP and INC routes, respectively. Higher doses of lidocaine had been administered in dogs, combining IP (8.8 mg kg^-1^) and INC (2 mg kg^-1^) routes; the doses studied achieved plasmatic levels of lidocaine well below toxic limits and no adverse effects were observed in dogs up to 18 hours after administration [32]. To the authors’ knowledge, there are no studies reporting the maximum recommended doses for IP and INC ropivacaine administration in dogs. However, in animal models, it is reported that ropivacaine has delayed cardiotoxic and neurotoxic side effects and a wider margin of safety compared to bupivacaine at equipotent doses [19]; in dogs undergoing ovariohysterectomy, Carpenter et al. (2004) did not observe any side effects with a combination of 4.4 mg kg^-1^ of IP and 2 ml of INC 0.75% bupivacaine. Furthermore, Lambertini et al. (2018) did not observe any adverse effects with 3 mg kg^-1^ of IP ropivacaine in dogs.

In this study, the efficacy of both IP and INC lidocaine and ropivacaine in reducing postoperative pain in dogs undergoing major abdominal surgeries was represented by significantly lower postoperative SF-GCMPS and VAS scores compared with baseline. No significant variations in SF-GCMPS and VAS scores between baseline and other postoperative examined periods were registered in dogs assigned to control group. However, there was a progressive and not statistically significant decrease in pain scores and this finding allows authors to suppose that even the combination between dexmedetomidine, methadone and meloxicam resulted in some beneficial effects in terms of postoperative pain relief. In fact, considering the time elapsed between premedication and extubation in the present study, methadone could still exert its analgesic effect two hours after extubation. It is also possible that the multimodal approach may have contributed to the postoperative pain scores reduction observed in L and R groups. Furthermore, pain assessment could have been impaired by the profound degree of sedation registered in the first hour after extubation; the dogs’ reaction to palpation of the surgical site could have been affected by sedation, decreasing the pain scores in L, R and C groups. This study revealed a significant difference in the need for postoperative rescue methadone between dogs that received IP and INC ropivacaine (1/10) and dogs in the control group (8/10). Although the number of postoperative rescue analgesia administrations did not significantly differ between groups L (5/11) and R (1/10), 4/5 dogs in L group received postoperative methadone from 9 hours after extubation; furthermore, SF-GCMPS scores did not significantly differ between the two groups from T0.5 to T9 and VAS score from T0.5 to T6, suggesting a comparable analgesic effect of the two treatments for 6 hours after extubation. Thereafter, in R group, postoperative SF-GCMPS scores were significantly lower than in groups L and C, at T12, T18 and T24, while postoperative VAS scores were significantly lower at T12; these results suggested a longer duration of the postoperative analgesic effect of IP and INC ropivacaine than IP and INC lidocaine. These results differ to those obtained in a previous study, where dogs receiving IP and INC lidocaine had significantly higher postoperative VAS scores compared to those obtained in dogs receiving IP and INC bupivacaine only at 0.5 hours after extubation [13]; therefore, results of the present study suggest a longer action of IP and INC ropivacaine compared with bupivacaine. Considering the lower postoperative SF-GCMPS and VAS scores and the number of postoperative rescue methadone administrations, the early onset of postoperative food intake for dogs in group R was unsurprising. These results confirm the benefit of optimal pain management in minimizing the requirement for postoperative analgesics and promoting earlier food intake. Our findings support the conclusion of another study, where the use of peripheral nerve block in dogs undergoing tibial plateau levelling osteotomy promoted lower requirement for postoperative methadone and a consequent greater postoperative food intake compared with a group of dogs treated with systemic analgesia [26].

This study has several limitations, one of which is that dogs had different abdominal pathologies and underwent different abdominal surgeries, with various degrees of inflammation and preoperative pain. These aspects could have affected the drugs’ absorption from IP and INC sites into the bloodstream and, consequently, their systemic and local analgesic effects. Furthermore, assessment of postoperative abdominal pain in small animals is subjective and lacks gold standard; in our study, in order to limit subjectivity and to increase the reliability of pain evaluation, two different pain scales were used, and pain was assessed by the same trained investigator, who was unaware of treatment allocation. Another limitation of the study is that the dilution of lidocaine and ropivacaine with 0.9% sterile saline to 5 mL kg^-1^ may have changed the physicochemical properties of both drugs [41] and consequently altered local anaesthetics onset of action, duration and efficacy.

## Conclusions

In conclusion, this study demonstrated that, as part of a multimodal approach to postoperative pain management for dogs undergoing major abdominal surgeries, IP and INC lidocaine and ropivacaine can provide effective and comparable post-surgical pain relief for 6 hours after extubation. In accordance with what has been previously hypothesized, IP and INC ropivacaine provided a longer lasting analgesic effect (up to 24 hours after extubation) than lidocaine and this finding results in a decreased postoperative opioids requirement and in early onset of food intake, compared with dogs in L and C groups. Therefore, IP and INC ropivacaine is recommended for postoperative pain management of dogs undergoing major abdominal surgeries.
